# Year 2 of Affordable Care Act Qualified Health Plans (QHPs) in a Medicaid Nonexpansion State: QHPs Associated With Viral Suppression for Virginia AIDS Drug Assistance Program Clients

**DOI:** 10.1093/ofid/ofy283

**Published:** 2018-10-31

**Authors:** Kathleen A McManus, Anne Rhodes, Lauren Yerkes, Carolyn L Engelhard, Karen S Ingersoll, Rebecca Dillingham

**Affiliations:** 1 Division of Infectious Diseases and International Health, University of Virginia, Charlottesville, Virginia; 2 Center for Health Policy, University of Virginia, Charlottesville, Virginia; 3 Virginia Department of Health, Richmond, Virginia; 4 Department of Public Health Sciences, University of Virginia, Charlottesville, Virginia; 5 Department of Psychiatry and Neurobehavioral Sciences, University of Virginia, Charlottesville, Virginia

**Keywords:** AIDS Drug Assistance, Program, health care reform, HIV, insurance, health, Patient Protection and Affordable Care Act

## Abstract

**Background:**

For year 1 of the Affordable Care Act (ACA), Virginia AIDS Drug Assistance Program (ADAP) clients with Qualified Health Plans (QHPs) achieved a higher rate of viral suppression. This study characterizes the demographic and health care delivery factors associated with QHP enrollment in year 2 and assesses the relationship between 2015 QHP coverage and HIV viral suppression.

**Methods:**

The cohort included Virginia ADAP clients who were eligible for ADAP-funded QHPs. Data were collected from 2014 to 2015. Multivariable binary logistic regression was conducted to assess the association of demographic and health care delivery factors with QHP enrollment and viral suppression.

**Results:**

In year 2, 63% of the cohort (n = 4631) enrolled in QHPs; 2015 ADAP-funded QHP enrollment was associated with 2014 ADAP-funded QHP (adjusted odds ratio [aOR], 111.11; 95% confidence interval [CI], 90.91–166.67), 2014 engagement in care (aOR, 2.16; 95% CI, 1.65–2.82), age (*P* < .001), race/ethnicity (*P* = .03), financial status (*P* < .001), and region (*P* < .001). For clients engaged in care (n = 2501), viral suppression was higher (83.3%) for those with ADAP-funded QHP coverage than for those who received medications from ADAP (79.9%). In multivariable binary logistic regression, achieving viral suppression was associated with 2015 QHP coverage (aOR, 1.27; 95% CI, 1.01–1.60), an initially undetectable viral load (aOR, 2.69; 95% CI, 2.13–3.39), gender (*P* = .03), age (*P* = .01), no AIDS diagnosis (aOR, 1.41; 95% CI, 1.12–1.78), financial status (*P* = .004), and region (*P* < .001).

**Conclusions:**

Virginia ADAP client 2015 QHP enrollment increased compared with year 1 and varied based on demographic and health care delivery factors. QHP coverage was again associated with viral suppression, an essential outcome for individuals and for public health.

In the United States, state AIDS Drug Assistance Programs (ADAPs) are a safety net that provides antiretroviral therapy (ART) for almost 260 000 low-income people living with HIV (PLWH), almost half of the PLWH in care in the United States [1, 2]. This program spends >$2 billion each year [1]. ADAPs are funded through federal and state mechanisms, and as a portion of Part B of the Ryan White HIV/AIDS Program, they are administered by the Health Resources and Services Administration (HRSA). Previously, the main health care delivery mechanism of state ADAPs was the direct provision of medication (Direct ADAP), with clients going to local health departments or designated pharmacies to pick up medications or state health departments mailing medications to clients’ homes. Since at least 2002, state ADAPs have purchased insurance for clients as the 1 cost-effective way to provide ART, and this practice has increased with the enactment of the Patient Protection and Affordable Care Act (ACA) [1]. HRSA released guidance supporting the practice in 2013 and 2014 [3, 4].

ADAPs’ goal is to provide cost-effective ART to as many low-income PLWH as possible. As recently as 2014, some state ADAPs had cost containment strategies, including waitlists, because they could not fund ART for all low-income PLWH who needed it [2]. Achieving viral suppression has important implications for an individual’s longevity and for HIV prevention in the community [5–8]. Additionally, each new HIV case prevented averts >$400 000 in annual health care costs [9].

When the ACA was enacted and first implemented, there was considerable debate about whether the provisions would improve US HIV care [10, 11]. There have been concerns about discriminatory practices in health insurance plans and prescription benefits [12]. For the 2014 ACA open enrollment period, the Virginia ADAP incorporated purchasing Qualified Health Plans (QHPs) into their health care delivery model [13]. They encouraged clients to enroll in QHPs and paid insurance premiums, deductibles, and medication copayments under the ACA’s federal health insurance marketplace [13].

Previously, we described the first year of the Virginia ADAP’s incorporation of the ACA into its health care delivery model [14]. We found that ADAP-funded QHP enrollment was associated with both demographic and health care delivery factors. Most importantly, Virginia ADAP clients who were engaged in care throughout the 2-year period and enrolled in ADAP-funded QHPs were more likely to achieve viral suppression than those who were engaged in care and received medications through Direct ADAP. To investigate further, we examined what happens over time to these associations, specifically whether the association between ADAP-funded QHP coverage and viral suppression was sustained. This study’s objective was to characterize the demographic and health care delivery factors associated with Virginia ADAP clients’ ADAP-funded QHP enrollment for year 2 and to assess whether the positive association between QHP coverage and HIV viral suppression remained in year 2 of the Virginia ADAP’s incorporation of the ACA.

## METHODS

**Figure 1.**
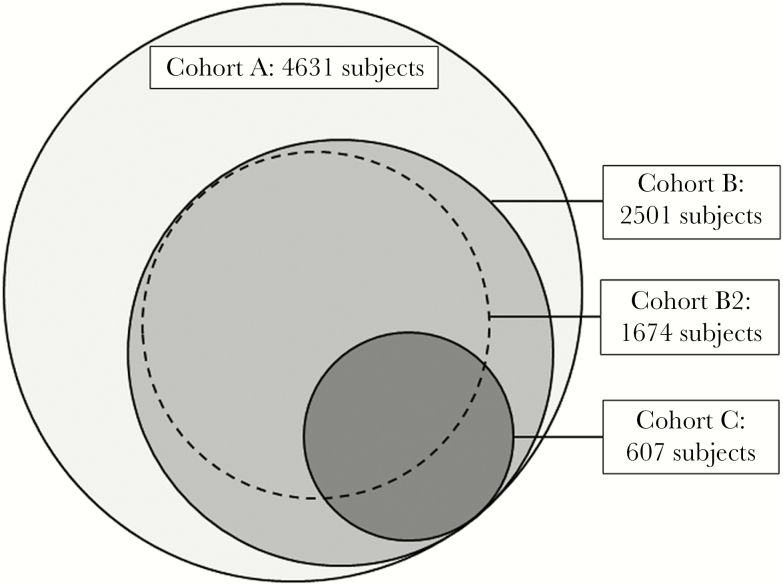
Description of nested-cohorts Virginia AIDS Drug Assistance Program (ADAP) clients were included in Cohort A if they were Virginia ADAP clients who were eligible for ADAP-funded Qualified Health Plans (QHPs), were between the ages of 18 and 64 years by January 1, 2014, were enrolled in ADAP by July 1, 2014, did not qualify for or have Medicare, and had a Social Security Number. Cohort A includes 4631 clients. Cohort B includes 2501 clients; it includes of members of Cohort A who demonstrate consistent engagement in care, as defined by at least 1 HIV viral load recorded in 2014 and at least 1 between July 1, 2015, and December 31, 2015. If an ADAP client had more than 1 viral load during the 6-month follow-up period, the last 1 was used for analysis. Cohort B2 includes 1674 clients; it includes members of Cohort B who had an ADAP-funded QHP during year 2 of the Affordable Care Act (2015). Cohort C includes 607 clients; it includes members of Cohort B who had an initially detectable viral load in 2014, indicating suboptimally controlled HIV disease.

The current methods are similar to the ones used in an analysis of year 1 of the Virginia ADAP’s incorporation of the ACA [14]. Four nested populations of interest were assessed in the study (Figure 1). Cohort A included all PLWH who were 18 to 64 years old on January 1, 2014, were ADAP clients by July 1, 2014, and had a Social Security Number. Only those with a Social Security Number are eligible for QHPs through the ACA. Cohort B included members of Cohort A who demonstrated consistent engagement in care in 2014 and 2015, as defined by at least 1 HIV viral load between January 1, 2014, and December 31, 2014, and at least 1 HIV viral load between July 1, 2015, and December 31, 2015. Cohort B2 included members of Cohort B with 2015 QHP coverage, and Cohort C included members of Cohort B who had an initially detectable viral load in 2014, indicating suboptimally controlled HIV disease. Data for all clients were de-identified and coded by the Virginia Department of Health (VDH) before transmission of the data set to researchers at the University of Virginia. The University of Virginia Institutional Review Board determined that the study was not human subjects research.

Data collected included age as of January 1, 2014, self-reported gender, race/ethnicity, HIV/AIDS diagnosis based on Centers for Disease Control and Prevention criteria using VDH HIV surveillance data [15], baseline CD4 count, and time since HIV diagnosis. All viral load dates and values were collected from January 1, 2014, through December 31, 2015. A client was categorized as engaged in HIV care in 2014 if they had at least 1 viral load in 2014. Health care delivery factors that were collected included 2014 ADAP program (ADAP-funded QHP or Direct ADAP), financial status (household income as percentage of the Federal Poverty Level), region of residence categorized into 1 of the 5 VDH health planning regions (Supplementary Figure 1), and presence of certified application counselors at the client’s HIV clinic during the ACA open enrollment period. As financial status affects eligibility for a tax credit (which subsidizes QHP costs and affects the cost of QHP coverage for ADAP), financial status was considered a health care delivery factor.

The 2 primary outcomes assessed include 2015 ADAP-funded QHP enrollment and viral load outcome. A client who maintained or achieved viral suppression in 2015, as defined as their last viral load in 2015 being ≤200 HIV RNA copies/mL, was categorized as having a good viral outcome, indicating viral suppression [16]. A poor viral outcome or viral failure was defined as the last viral load in 2015 being >200 HIV RNA copies/mL.

The percentage of Cohort A that enrolled in ADAP-funded QHP was calculated for the cohort and for demographic and health care delivery groups. Univariable binary regression and multivariable binary logistic regression were used to determine the strength and significance of the association between characteristics and ADAP-funded QHP enrollment. The percentage of Cohorts B and C that achieved viral suppression was assessed for each cohort and then for demographic and health care delivery groups. Univariable and multivariable binary logistic regression were used to determine the strength and significance of the association between characteristics and viral suppression.

As a secondary analysis to examine who was not achieving good outcomes despite health insurance, the percentage of Cohort B2 that had viral failure was determined for the cohort and for characteristic and health care delivery groups. Univariable and multivariable binary logistic regression were used to determine the association between characteristics and viral failure. As an additional secondary analysis, Cohort C’s achievement of viral suppression was analyzed in a similar manner to Cohort B. All statistical analyses were conducted using IBM SPSS Statistics software, version 24.

## RESULTS

Table 1 presents characteristics of the nested cohorts: A (n = 4631), B (n = 2501), B2 (n = 1674), and C (n = 607). Twelve clients eligible for Cohort A were missing baseline characteristic data; given that they represented less than 1% of the sample, they were not included. For year 2, 63% of Cohort A enrolled in ADAP-funded QHPs. Table 2 presents enrollment rates for each characteristic or factor category and unadjusted and adjusted odds ratios for the association between QHP enrollment and client and health care delivery characteristics. Multivariable regression, controlling for gender, HIV/AIDS diagnosis, and the presence of clinic-located certified application counselors, demonstrates that 2015 ADAP-funded QHP enrollment was associated with age (*P* < .001), race/ethnicity (*P* = .03), 2014 engagement in care (*P* < .001), 2014 ADAP-funded QHP coverage (*P* < .001), financial status (*P* < .001), and region (*P* < .001).

**Table 1.** Characteristics of Cohorts

Characteristic/Factor	Cohort A, No. (Col %)	Cohort B, No. (Col %)	Cohort B2, No. (Col %)	Cohort C, No. (Col %)
All	4631	2501	1674	607
Age, y				
18–24	307 (6.6)	149 (6.0)	105 (6.3)	60 (9.9)
25–34	951 (20.5)	465 (18.6)	325 (19.4)	149 (24.5)
35–44	1013 (21.9)	566 (22.6)	394 (23.5)	139 (22.9)
45–54	1575 (34.0)	873 (34.9)	587 (35.1)	197 (32.5)
55–64	785 (17.0)	448 (17.9)	263 (15.7)	62 (10.2)
Race/ethnicity				
American Indian/Alaska Native/Native Hawaiian	33 (0.7)	19 (0.8)	14 (0.8)	5 (0.8)
Asian	79 (1.7)	41 (1.6)	33 (2.0)	7 (1.2)
Black/African American	2968 (64.1)	1595 (63.8)	1075 (64.2)	429 (70.7)
Hispanic/Latino	318 (6.9)	197 (7.9)	133 (7.9)	34 (5.6)
White	1223 (26.6)	649 (25.9)	419 (25.0)	132 (21.7)
Gender				
Female	1220 (26.3)	716 (28.6)	498 (29.7)	169 (27.8)
Transgender	48 (1.0)	18 (0.7)	12 (0.7)	5 (0.8)
Male	3363 (72.6)	1767 (70.7)	1164 (69.5)	433 (71.3)
HIV/AIDS diagnosis				
AIDS diagnosis	1570 (33.9)	923 (36.9)	581 (34.7)	219 (36.1)
HIV diagnosis	3061 (66.1)	1578 (63.1)	1093 (65.3)	388 (63.9)
2014 ADAP program				
ADAP-funded QHP	2330 (50.3)	1306 (52.2)	1674 (100)	267 (44.0)
Direct ADAP	2301 (49.7)	1195 (47.8)	0 (0)	340 (56.0)
Financial status				
251%–400% FPL (tax credit)	349 (7.5)	221 (8.8)	119 (7.1)	45 (7.4)
139%–250% FPL (tax credit)	1034 (22.3)	590 (23.6)	335 (20.0)	125 (20.6)
101%–138% FPL (Medicaid gap with tax credit)	571 (12.3)	300 (12.0)	225 (13.4)	51 (8.4)
<100% FPL (Medicaid gap, no tax credit)	2677 (57.8)	1390 (55.6)	995 (59.4)	386 (63.6)
Region of residence				
Northwest	473 (10.2)	322 (12.9)	530 (31.7)	42 (6.9)
Eastern	1562 (33.7)	481 (19.2)	317 (18.9)	244 (40.2)
Central	1074 (23.2)	667 (26.7)	437 (26.1)	133 (21.9)
Southwest	511 (11.0)	324 (13.0)	177 (10.6)	72 (11.9)
Northern	1011 (21.8)	707 (28.3)	530 (31.7)	116 (19.1)
Average CD4 count, cells/mm^3^	579 ± 321^1^	579 ± 321^3^	592 ± 313^5^	437 ± 331^7^
Average time since HIV diagnosis, y	10.3 ± 7.1^2^	10.5 ± 7.1^4^	10.1 ± 6.9^6^	9.4 ± 7.1^8^

Cohort A included all people living with HIV (PLWH) who were 18 to 64 years old on January 1, 2014, were AIDS Drug Assistance Program (ADAP) clients by July 1, 2014, did not have Medicare, and had a Social Security Number. Cohort B included members of Cohort A who demonstrated consistent engagement in care in 2014 and 2015, as defined by at least 1 HIV viral load recorded in 2014 and at least 1 HIV viral load between July 1, 2015, and December 31, 2015. Cohort B2 included members of Cohort B with 2015 ADAP-funded Qualified Health Plan (QHP) coverage. Cohort C included members of Cohort B who had an initially detectable viral load in 2014, indicating suboptimally controlled HIV disease.

(1) Data available for 4363 subjects in Cohort A. (2) Data available for 4064 subjects in Cohort A. (3) Data available for 2469 subjects in Cohort B. (4) Data available for 2312 subjects in Cohort B. (5) Data available for 1671 subjects in Cohort B2. (6) Data available for 1537 subjects in Cohort B2. (7) Data available for 605 subjects in Cohort C. (8) Data available for 561 subjects in Cohort C.

Abbreviations: ADAP, AIDS Drug Assistance Program; FPL, Federal Poverty Level; QHP, Qualified Health Plan.

**Table 2.** Year 2 Affordable Care Act QHP Enrollment of Virginia ADAP Clients who Were Eligible for ADAP-Funded QHPs (Cohort A): Frequencies and Results of Univariable and Multivariable Binary Logistic Regression

Characteristic/Factor	Enrollment, No. (Row %)	Unadjusted OR (95% CI)	*P* Value	Adjusted OR (95% CI)	*P* Value
All	2916 (63.0)	NA	NA	NA	NA
Age, y			<.001		<.001
18–24	197 (64.2)	1.46 (1.11–1.92)		1.70 (1.12–2.58)	
25–34	600 (63.1)	1.40 (1.15–1.69)		1.75 (1.28–2.38)	
35–44	677 (66.8)	1.65 (1.36–2.00)		1.97 (1.46–2.65)	
45–54	1010 (64.1)	1.46 (1.23–1.74)		1.66 (1.26–2.19)	
55–64	432 (55.0)	Reference		Reference	
Race/ethnicity			.01		.03
American Indian/Alaska Native/Native Hawaiian	26 (78.8)	2.48 (1.07–5.77)		2.34 (0.73–7.47)	
Asian	58 (73.4)	1.85 (1.11–3.08)		2.43 (1.22–4.83)	
Black/African American	1896 (63.9)	1.09 (0.85–1.40)		1.28 (1.02–1.61)	
Hispanic/Latino	197 (61.9)	1.18 (1.03–1.36)		1.04 (0.71–1.54)	
White	739 (59.9)	Reference		Reference	
Gender			.001		.1
Female	821 (67.3)	1.29 (1.13–1.49)		1.25 (1.02–1.53)	
Transgender	30 (62.5)	1.05 (0.58–1.89)		1.22 (0.55–2.70)	
Male	2065 (61.4)	Reference		Reference	
HIV/AIDS diagnosis			<.001		.1
HIV diagnosis	1989 (65.0)	1.29 (1.14–1.46)		1.17 (0.97–1.42)	
AIDS diagnosis	927 (59.0)	Reference		Reference	
Engaged in care in 2014			<.001		<.001
Viral load present	2510 (64.5)	1.48 (1.27–1.74)		2.16 (1.65–2.82)	
Viral load absent	406 (55.0)	Reference		Reference	
2014 ADAP program			<.001		<.001
ADAP-funded QHP	2270 (97.4)	96.93 (73.84–127.22)		111.11 (90.91–166.67)	
Direct ADAP	646 (28.1)	Reference		Reference	
Financial status			<.001		<.001
251%–400% FPL (tax credit)	189 (54.2)	0.61 (0.49–0.77)		0.58 (0.41–0.82)	
139%–250% FPL (tax credit)	589 (57.0)	0.69 (0.59–0.80)		0.64 (0.51–0.81)	
101%–138% FPL (Medicaid gap with tax credit)	376 (65.8)	1.00 (0.82–1.21)		0.94 (0.70–1.25)	
<100% FPL (Medicaid gap, no tax credit)	1762 (65.8)	Reference		Reference	
Region of residence			<.001		<.001
Northwest	305 (64.5)	0.83 (0.66–1.04)		0.36 (0.25–0.54)	
Eastern	1005 (64.3)	0.82 (0.70–0.98)		0.61 (0.48–0.79)	
Central	651 (60.6)	0.70 (0.59–0.84)		0.51 (0.37–0.70)	
Southwest	261 (51.1)	0.48 (0.38–0.59)		0.58 (0.42–0.79)	
Northern	694 (68.6)	Reference		Reference	
Clinic-located CAC			.001		.8
Present	1695 (65.1)	1.24 (1.10–1.39)		1.02 (0.82–1.28)	
Absent	1221 (60.2)	Reference		Reference	

Abbreviations: ADAP, AIDS Drug Assistance Program; CAC, Certified Application Counselor; CI, confidence interval; FPL, Federal Poverty Level; QHP, Qualified Health Plan.

For client-level characteristics, all age groups were more likely to enroll than the reference group, age 55–64 years (18–24 years: adjusted odds ratio [aOR], 1.70; 95% confidence interval [CI], 1.12–2.58; 25–34 years: aOR, 1.75; 95% CI, 1.28–2.38; 35–44 years: aOR, 1.97; 95% CI, 1.46–2.65; 45–54 years: aOR, 1.66; 95% CI, 1.26–2.19). For race/ethnicity, black/African American (aOR, 1.28; 95% CI, 1.02–1.61) and Asian (aOR, 2.43; 95% CI, 1.22–4.83) clients were more likely to enroll than other race/ethnicity groups. Those who demonstrated engagement in care in 2014 were more than twice as likely to enroll as those without engagement in 2014 (aOR, 2.16; 95% CI, 1.65–2.82). For health care delivery factors, 97.4% of those who had 2014 ADAP-funded QHP coverage opted for 2015 ADAP-funded QHP enrollment, compared with only 28.1% of those who had Direct ADAP in 2014 (aOR, 111.11; 95% CI, 90.91–166.67). Regarding financial status, clients who were 139%–250% FPL (aOR, 0.64; 95% CI, 0.51–0.81) and 250%–400% FPL (aOR, 0.58; 95% CI, 0.41–0.82) were less likely to enroll compared with clients below 139% FPL. Clients residing in the Northern VDH planning region enrolled at the highest rate (68.6%), with clients in all other regions being less likely to enroll (Northwest: aOR, 0.36; 95% CI, 0.25–0.54; Eastern: aOR, 0.61; 95% CI, 0.48–0.79; Central: aOR, 0.51; 95% CI, 0.37–0.70; Southwest: aOR, 0.58; 95% CI, 0.42–0.79).

For Cohort B (n = 2501), 66.9% were enrolled in QHPs, and the remaining portion accessed ART through Direct ADAP. In Cohort B, ADAP clients with QHP coverage had a higher rate of viral suppression, at 83.3%, compared with 79.9% for those who remained on Direct ADAP. Table 3 presents rates of viral suppression for each characteristic or factor category, and it also presents the results of binary logistic regression, as well as unadjusted and adjusted odds ratios for the associations with viral suppression. In multivariable binary logistic regression, controlling for time and race/ethnicity, achieving viral suppression was associated with age (*P* = .01), gender (*P* = .03), HIV without progression to AIDS (*P* = .004), an initially undetectable viral load (*P* < .001), 2015 ADAP-funded QHP coverage (*P* = .04), financial status (*P* = .004), and region (*P* < .001).

**Table 3.** Viral Suppression Outcomes of Virginia AIDS Drug Assistance Program Clients who Demonstrated Engagement in Care in 2014 and 2015 (Cohort B): Frequencies and Results of Univariable and Multivariable Binary Logistic Regression

Characteristic/Factor	Good Viral Outcome, No. (Row %)	Unadjusted OR (95% CI)	*P* Value	Adjusted OR (95% CI)	*P* Value
All	2056 (82.2)	NA	NA	NA	NA
2015 ADAP program			.04		.04
ADAP-funded QHP	1395 (83.3)	1.25 (1.02–1.55)		1.27 (1.01–1.60)	
Direct ADAP	661 (79.9)	Reference		Reference	
Initial viral suppression (2014)			<.001		<.001
Undetectable	1660 (87.6)	3.78 (3.05–4.69)		2.69 (2.13–3.39)	
Detectable	396 (65.2)	Reference		Reference	
Age, y			<.001		.01
18–24	110 (73.8)	0.36 (0.23–0.58)		0.52 (0.31–0.87)	
25–34	354 (76.1)	0.41 (0.29–0.59)		0.53 (0.36–0.78)	
35–44	467 (82.5)	0.61 (0.42–0.87)		0.75 (0.51–1.10)	
45–54	728 (83.4)	0.65 (0.46–0.91)		0.75 (0.53–1.07)	
55–64	397 (88.6)	Reference		Reference	
Race/ethnicity			.06		.5
American Indian/Alaska Native/Native Hawaiian	18 (94.7)	3.47 (0.46–26.31)		6.57 (0.83–52.22)	
Asian	36 (87.8)	1.39 (0.53–3.62)		1.12 (0.41–3.04)	
Black/African American	1287 (80.7)	0.81 (0.63–1.03)		1.09 (0.83–1.44)	
Hispanic/Latino	171 (86.8)	1.27 (0.80–2.02)		1.19 (0.72–1.96)	
White	544 (83.8)	Reference		Reference	
Gender			.03		.03
Female	578 (80.7)	0.86 (0.69–1.07)		0.79 (0.62–1.02)	
Transgender	11 (61.1)	0.32 (0.12–0.87)		0.32 (0.11–0.91)	
Male	1467 (83.0)	Reference		Reference	
HIV/AIDS diagnosis			.2		.004
HIV diagnosis	1308 (82.9)	1.13 (0.92–1.40)		1.41 (1.12–1.78)	
AIDS diagnosis	748 (81.0)	Reference		Reference	
Financial status			<.001		.004
251%–400% FPL (tax credit)	198 (89.6)	2.30 (1.47–3.61)		1.97 (1.23–3.17)	
139%–250% FPL (tax credit)	508 (86.1)	1.66 (1.27–2.16)		1.51 (1.30–2.00)	
101%–138% FPL (Medicaid gap with tax credit)	253 (84.3)	1.44 (1.03–2.01)		1.20 (0.84–1.72)	
<100% FPL (Medicaid gap, no tax credit)	1097 (78.9)	Reference		Reference	
Region of residence			<.001		<.001
Northwest	285 (88.5)	1.10 (0.73–1.65)		1.13 (0.74–1.74)	
Eastern	309 (64.2)	0.26 (0.19–0.34)		0.40 (0.29–0.55)	
Central	569 (85.3)	0.83 (0.61–1.13)		0.99 (0.71–1.38)	
Southwest	274 (84.6)	0.78 (0.54–1.13)		0.95 (0.63–1.42)	
Northern	619 (87.6)	Reference		Reference	
Days observed			<.001		.3

Abbreviations: ADAP, AIDS Drug Assistance Program; CI, confidence interval; FPL, Federal Poverty Level; QHP, Qualified Health Plan.

Clients aged 18–24 years (aOR, 0.52; 95% CI, 0.31–0.87) and 25–34 years (aOR, 0.53; 95% CI, 0.36–0.78) were less likely to achieve viral suppression than those over the age of 34. Transgender clients (aOR, 0.32; 95% CI, 0.11–0.91) were less likely than cis-gender male or female clients to achieve viral suppression. Clients who had not progressed to an AIDS diagnosis were more likely to have viral suppression (aOR, 1.41; 95% CI, 1.12–1.78). Having an initially undetectable viral load was associated with viral suppression (aOR, 2.69; 95% CI, 2.13–3.39). Additionally, having an ADAP-funded QHP was also associated with viral suppression compared with being on Direct ADAP (aOR, 1.27; 95% CI, 1.01–1.60). Clients who had incomes in the 139%–250% FPL (aOR, 1.51; 95% CI, 1.30–2.00) and 250%–400% FPL (aOR, 1.97; 95% CI, 1.23–3.17) range were more likely to achieve viral suppression compared with clients below 139% FPL. Clients in the Eastern VDH planning region had a lower likelihood of achieving viral suppression compared with the other 4 regions (aOR, 0.40; 95% CI, 0.29–0.55).

For Cohort B2 (n = 1674), 16.7% of clients had viral failure despite being supported with a 2015 ADAP-funded QHP. Supplementary Table 1 presents rates of viral failure for each characteristic or factor category, and it also presents the results of binary logistic regression, as well as unadjusted and adjusted odds ratios for the associations with viral failure. In multivariable binary logistic regression, controlling for time, age, race/ethnicity, gender, HIV without progression to AIDS, and financial status, viral failure was associated with an initially detectable viral load (*P* < .001) and region (*P* < .001). A client with an initially detectable viral load in 2014 was more likely to have viral failure in 2015 (aOR, 2.66; 95% CI, 1.98–3.56), and clients in the Eastern region were more likely to have viral failure compared with clients residing in other regions (aOR, 2.57; 95% CI, 1.73–3.81).

For Cohort C (n = 607), 65.2% of clients who started with a detectable viral load in 2014 did achieve viral suppression in 2015. Supplementary Table 2 presents rates of good viral outcome for each characteristic or factor category, and it also presents the results of binary logistic regression, as well as unadjusted and adjusted odds ratios for the associations with viral suppression. No progression to AIDS (*P* < .001) and region of residence (*P* < .001) were not associated with viral suppression in multivariate logistic regression, controlling for 2015 ADAP program, age, race/ethnicity, gender, financial status, and time. Having a diagnosis of HIV rather than AIDS was associated with viral suppression (aOR, 1.99; 95% CI, 1.35–2.93). In terms of residence, clients in the Eastern VDH planning region had a lower likelihood of achieving viral suppression compared with the other 4 regions (aOR, 0.57; 95% CI, 0.34–0.97).

## DISCUSSION

**Figure 2.**
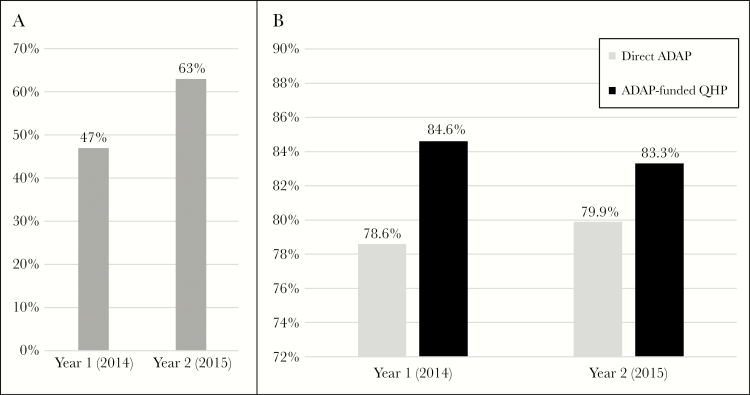
A, AIDS Drug Assistance Program (ADAP)–funded Qualified Health Plan (QHP) enrollment of Cohort A by year: Cohort A was defined in a similar way from year to year (see the “Methods” or Figure 1), but the size of Cohort A increased from year 1 (2014) to year 2 (2015). For year 1 of the Virginia ADAP’s incorporation of the Affordable Care Act (ACA) into their health care delivery model, 47.1% of a similar Virginia Cohort A were enrolled in ADAP-funded QHPs [14]. This percentage increased to 63% of Cohort A for year 2. B, Viral suppression for Cohort B (clients engaged in care) with direct ADAP or ADAP-funded QHP by year: Cohort B was defined in a similar way from year to year (see the “Methods” or Figure 1). For year 1 of the Virginia ADAP’s incorporation of the ACA into their health care delivery model, 78.6% of clients in a similar Virginia Cohort B achieved 2014 viral suppression compared with 84.6% of those clients who had 2014 ADAP-funded QHP coverage [14]. For year 2, 79.9% of clients in Cohort B achieved 2015 viral suppression compared with 83.3% of those clients who had 2015 ADAP-funded QHP coverage.

Compared with the national ADAP population in 2014, this study population is comparable to the national ADAP population, except for a greater proportion of clients aged 18–24 years old, who were African American, and who had incomes below 100% FPL [17]. For year 2, 63% of Cohort A enrolled in ADAP-funded QHPs compared with 47.1% of a similarly defined cohort for year 1 (Figure 2A) [14]. In 2015, about 52% of national ADAP clients had ADAP-funded health insurance [18]. During the second ACA open enrollment period, the VDH increased their state-coordinated communication with HIV clinics to reach ADAP clients who were not enrolled in ADAP-funded QHPs during year 1. Based on our year 1 and year 2 analyses, these efforts were successful and resulted in an increase in ADAP-funded QHP enrollment from 1853 to 2916, or 57% [14].

Similar to year 1, both demographic and health care delivery factors were associated with enrollment. All clients younger than 54 years and African Americans were more likely to enroll. These groups do not generally have higher engagement in care, and they were less likely to enroll in 2014 [14, 19–21]. We did not expect these groups to be more likely to enroll in 2015. It is possible that the state-coordinated communication with HIV clinics for the second open enrollment period resulted in more targeted outreach, but this unexpected finding will need to be investigated in future research. Being engaged in care in 2014 was associated with enrollment in QHPs. We hypothesized that this effect occurred because contact with the health care system is needed to know about the option and to enroll.

The strongest association with 2015 ADAP-funded QHP enrollment was enrollment in an ADAP-funded QHP in 2014, making clients more than 100 times as likely to enroll in 2015. One explanation for this it that from 2014 to 2015, there were not significant changes in carrier coverage, and many ADAP clients were auto-enrolled in ADAP-funded QHPs. Additionally, it is possible that those who were on Direct ADAP in 2014 and stayed on Direct ADAP were undocumented immigrants who were ineligible for ACA Marketplace insurance plans or that this group who remained on Direct ADAP had other social determinants of health (such as mental health issues, substance use, unstable housing, lack of transportation, lack of computer/Internet access, or food insecurity) or language barriers that negatively impacted their ability to complete the enrollment process.

During the first year of enrollment, VDH prioritized those with incomes >139% FPL because they were the most cost-effective to enroll; this resulted in increased enrollment for these clients [13]. For year 2, there was no such state-led prioritization, and the data demonstrate that those with incomes <139% FPL were more likely to enroll. Surprisingly, co-located Certified Application Counselors at HIV clinics were not associated with increased rates of enrollment. Region was a factor in terms of which clients enrolled. It is likely that regional differences in resources for PLWH affected enrollment rates. Future studies should examine geographical differences in care-related resources in greater detail.

For Cohort B, clients who engaged in care in 2014 and 2015, a viral suppression rate of 82.2% was achieved. This is higher than the national estimated average of 49% viral suppression for all PLWH in the United States and is similar to viral suppression rates of 73%–75% for Ryan White HIV/AIDS Program clinics [20, 22–24]. This cohort’s viral suppression rate is slightly lower than the HRSA’s HIV/AIDS Bureau’s national and Virginia data for 2015, which demonstrates that 83.4% and 86.4% of PLWH who had at least 1 Ryan White HIV/AIDS Program–funded outpatient ambulatory health services visit in 2016 and at least 1 HIV viral load in 2016 achieved viral suppression [25].

In examining the effect of ADAP-funded QHPs on viral suppression for Cohort B for year 2, the odds of achieving viral suppression were 27% higher for clients who had 2015 ADAP-funded QHP coverage compared with those with 2015 Direct ADAP. Cohort B was defined in a similar way from year to year, and compared with year 1, the absolute difference in viral suppression between ADAP-funded QHPs and Direct ADAP decreased from 6.0% to 3.4% (Figure 2B).

Similar to national data, younger age and transgender clients were less likely to achieve viral suppression [25]. Unfortunately, for clients who did not have optimal control of their HIV at the beginning of 2014 but who engaged in care in 2014 and 2015 (Cohort C), ADAP-funded QHP coverage was not associated with achieving viral suppression. This is different than what was demonstrated for year 1 [14]. Obtaining insurance was not sufficient for these clients to achieve viral suppression. We do not have data on this cohort’s access to and use of Ryan White services before and after QHP enrollment; this is an area for future investigation.

For Cohorts B, B2, and C, the Eastern region was associated with a lower likelihood of viral suppression. The Eastern region has a disproportionate share of Virginia’s HIV/AIDS epidemic. The Virginia Beach-Norfolk-Newport News Metropolitan Statistical Area is home to 21% of the Virginia population and 31% of Virginians living with HIV. In this region, up to 33% of PLWH are considered out of care [26]. The interaction of geography and the distribution of resources, including federal Ryan White funding, Ryan White clinics, and/or HIV medical providers, are topics that need exploration in future work, in addition to contributions of other social determinants of health or potential data reporting issues. Additionally, until 2016, there were issues with electronic viral load reporting from the largest lab in the Eastern region. We are hopeful that with more complete reporting, the viral suppression rate may increase, and we plan to assess this in future analyses.

As with any study, there are limitations. There could be unmeasured differences in social determinants of health, such as education, housing stability, or food security, between those who enrolled in ADAP-funded QHPs and those who continued to receive medications from Direct ADAP. Unfortunately, it is difficult to obtain statewide client-level data that include those variables. Moreover, it is possible that using the final viral load value to define viral suppression could incorrectly estimate viral suppression in our cohort [27]. Additionally, in 2015, electronic lab reporting had not yet been implemented for all labs in the state, which may have affected the availability of viral load data, especially in the Eastern region.

Future studies should include additional Medicaid nonexpansion states and Medicaid expansion states, so that post-ACA HIV outcomes can be understood on a larger scale. Additionally, incorporating more data about Ryan White HIV/AIDS Program services received at the client level would allow for a better understanding of the impact of the interaction between health care insurance and Ryan White HIV care. The comprehensive and wraparound services provided by Ryan White clinics, such as substance use counseling, mental health services, case management, nutrition support, and transportation, may not be covered by insurance. For year 2 of the ACA, ADAP-funded QHP coverage in Virginia was again associated with higher rates of viral suppression, an essential outcome for individuals and for public health [5–8]. With the end of the ACA’s individual mandate and the ongoing possibility of changes to the ACA and Medicaid, there are important lessons from this work related to defining the best HIV health care delivery models [28–31].

## Supplementary Data

Supplementary materials are available at *Open Forum Infectious Diseases* online. Consisting of data provided by the authors to benefit the reader, the posted materials are not copyedited and are the sole responsibility of the authors, so questions or comments should be addressed to the corresponding author.

ofy283_suppl_supplementary_tables

ofy283_suppl_supplementary_figure
