# Supplementary material for: Year 2 of Affordable Care Act Qualified Health Plans (QHPs) in a Medicaid Nonexpansion State: QHPs Associated With Viral Suppression for Virginia AIDS Drug Assistance Program Clients
Source: Open Forum Infect Dis. 2018 Oct 31;5(12):ofy283. doi: 10.1093/ofid/ofy283 (PMC6293482; doi:10.1093/ofid/ofy283)
Supplement: ofy283_suppl_supplementary_tables [file ofy283_suppl_supplementary_tables.docx]

**Supplemental Figure 1. Health planning regions of the Virginia Department of Health:** Virginia Department of Health has five planning regions demonstrated in the map: Central, Eastern, Northern, Northwest, and Southwest.

| **Supplemental Table 1. Poor Viral Outcomes of Virginia AIDS Drug Assistance Program (ADAP) Clients with ADAP-funded Qualified Health Plan Coverage (Cohort B2): frequencies and results of univariable and multivariable binary logistic regression** | | | | | | | | | | |  |
| --- | --- | --- | --- | --- | --- | --- | --- | --- | --- | --- | --- |
| **Characteristic/Factor** | | **Poor Viral Outcome n (row %)** | | **Unadjusted OR (95% CI)** | | **p value** | | **Adjusted OR (95% CI)** | | **p value** | |
| All | | 279 (16.7) | | NA | | NA | | NA | | NA | |
|  | |  | |  | |  | |  | |  | |
| **Initial Viral Suppression (2014)** | |  | |  | | **< 0.001** | |  | | **< 0.001** | |
| Detectable | | 126 (32.4) | | 3.55 (2.70-4.65) | |  | | 2.66 (1.98-3.56) | |  | |
| Undetectable | | 153 (11.9) | | Reference | |  | | Reference | |  | |
|  | |  | |  | |  | |  | |  | |
| **Age (years)** | |  | |  | | **0.03** | |  | | 0.1 | |
| 18–24 | | 27 (25.7) | | 2.26 (1.28-3.96) | |  | | 1.83 (0.99-3.37) | |  | |
| 25–34 | | 61 (18.8) | | 1.51 (0.96-2.37) | |  | | 1.31 (0.81-2.12) | |  | |
| 35–44 | | 57 (14.5) | | 1.10 (0.70-1.73) | |  | | 0.93 (0.57-1.49) | |  | |
| 45–54 | | 99 (16.9) | | 1.32 (0.87-2.00) | |  | | 1.22 (0.79-1.89) | |  | |
| 55–64 | | 35 (13.3) | | Reference | |  | | Reference | |  | |
|  | |  | |  | |  | |  | |  | |
| **Race/Ethnicity** | |  | |  | | 0.4 | |  | | 0.7 | |
| American Indian/ Alaska Native/ Native Hawaiian | | 1 (7.1) | | 0.40 (0.05-3.09) | |  | | 0.22 (0.03-1.79) | |  | |
| Asian | | 3 (9.1) | | 0.52 (0.15-1.74) | |  | | 0.68 (0.19-2.40) | |  | |
| Black/African American | | 190 (17.7) | | 1.11 (0.82-1.50) | |  | | 0.98 (0.70-1.37) | |  | |
| Hispanic/Latino | | 17 (12.8) | | 0.76 (0.43-1.34) | |  | | 0.93 (0.50-1.72) | |  | |
| White | | 68 (16.2) | | Reference | |  | | Reference | |  | |
|  | |  | |  | |  | |  | |  | |
| **Gender** | |  | |  | | 0.2 | |  | | 0.3 | |
| Female | | 90 (18.1) | | 1.17 (0.89-1.54) | |  | | 1.21 (0.89-1.64) | |  | |
| Transgender | | 4 (33.3) | | 2.65 (0.79-8.88) | |  | | 2.23 (0.57-8.72) | |  | |
| Male | | 185 (15.9) | | Reference | |  | | Reference | |  | |
|  | |  | |  | |  | |  | |  | |
| **HIV/AIDS Diagnosis** | |  | |  | | 0.3 | |  | | **0.02** | |
| AIDS Diagnosis | | 105 (18.1) | | 1.17 (0.89-1.52) | |  | | 1.40 (1.04-1.87) | |  | |
| HIV Diagnosis | | 174 (15.9) | | Reference | |  | | Reference | |  | |
|  | |  | |  | |  | |  | |  | |
| **Financial Status** | |  | |  | | 0.2 | |  | | 0.4 | |
| 251-400% FPL (Tax Credit) | | 13 (10.9) | | 0.57 (0.31-1.04) | |  | | 0.65 (0.35-1.22) | |  | |
| 139-250% FPL (Tax Credit) | | 50 (14.9) | | 0.82 (0.58-1.15) | |  | | 0.83 (0.58-1.19) | |  | |
| 101-138% FPL      (Medicaid Gap with Tax Credit) | | 40 (17.8) | | 1.01 (0.69-1.47) | |  | | 1.10 (0.74-1.65) | |  | |
| <100% FPL      (Medicaid Gap, no Tax Credit) | | 176 (17.7) | | Reference | |  | | Reference | |  | |
|  | |  | |  | |  | |  | |  | |
| **Region of Residence** | |  | |  | | **< 0.001** | |  | | **< 0.001** | |
| Northwest | | 27 (12.7) | | 1.02 (0.63-1.65) | |  | | 0.95 (0.58-1.58) | |  | |
| Eastern | | 108 (34.1) | | 3.63 (2.57-5.14) | |  | | 2.57 (1.73-3.81) | |  | |
| Central | | 49 (11.2) | | 0.89 (0.60-1.32) | |  | | 0.79 (0.52-1.21) | |  | |
| Southwest | | 29 (16.4) | | 1.38 (0.86-2.21) | |  | | 1.17 (0.70-1.95) | |  | |
| Northern | | 66 (12.5) | | Reference | |  | | Reference | |  | |
|  | |  | |  | |  | |  | |  | |
| **Days Observed** | |  | |  | | .001 | |  | | 0.4 | |
|  | |  | |  | |  | |  | |  | |
| Abbreviations: CI: Confidence Interval, HIV: Human Immunodeficiency Virus, AIDS: Acquired Immunodeficiency Syndrome, FPL: Federal Poverty Level. | | | | | | | | | | | |
| **Supplemental Table 2. Viral Suppression Outcomes of Virginia AIDS Drug Assistance Program Clients who demonstrated engagement in care in 2014 and 2015 and had initially detectable viral loads in 2014 (Cohort C):** frequencies and results of univariable and multivariable binary logistic regression | | | | | | | | | | |  |
| **Characteristic/Factor** | **Good Viral Outcome n (row %)** | | **Unadjusted OR (95% CI)** | | **p value** | | **Adjusted OR (95% CI)** | | **p value** | |  |
| All | 396 (65.2) | | NA | | NA | | NA | | NA | |  |
|  |  | |  | |  | |  | |  | |  |
| **2015 ADAP Program** |  | |  | | 0.1 | |  | | 0.1 | |  |
| ADAP-funded QHP | 263 (67.6) | | 1.33 (0.95-1.88) | |  | | 1.33 (0.92-1.94) | |  | |  |
| Direct ADAP | 133 (61.0) | | Reference | |  | | Reference | |  | |  |
|  |  | |  | |  | |  | |  | |  |
| **Age (years)** |  | |  | | 0.3 | |  | | 0.2 | |  |
| 18–24 | 40 (66.7) | | 0.64 (0.29-1.41) | |  | | 0.50 (0.21-1.18) | |  | |  |
| 25–34 | 95 (63.8) | | 0.56 (0.29-1.10) | |  | | 0.50 (0.25-1.03) | |  | |  |
| 35–44 | 94 (67.6) | | 0.67 (0.34-1.32) | |  | | 0.69 (0.33-1.41) | |  | |  |
| 45–54 | 120 (60.9) | | 0.50 (0.26-0.95) | |  | | 0.48 (0.24-0.95) | |  | |  |
| 55–64 | 47 (75.8) | | Reference | |  | | Reference | |  | |  |
|  |  | |  | |  | |  | |  | |  |
| **Race/Ethnicity** |  | |  | | 0.1 | |  | | 0.3 | |  |
| American Indian/ Alaska Native/Native Hawaiian | 4 (80.0) | | 1.87 (0.20-17.22) | |  | | 2.63 (0.26-26.55) | |  | |  |
| Asian | 5 (71.4) | | 1.17 (0.22-6.26) | |  | | 0.88 (0.15-5.14) | |  | |  |
| Black/African American | 268 (62.5) | | 0.78 (0.51-1.18) | |  | | 1.09 (0.69-1.73) | |  | |  |
| Hispanic/Latino | 29 (85.3) | | 2.71 (0.98-7.49) | |  | | 3.02 (1.03-8.91) | |  | |  |
| White | 90 (68.2) | | Reference | |  | | Reference | |  | |  |
|  |  | |  | |  | |  | |  | |  |
| **Gender** |  | |  | | 0.2 | |  | | 0.4 | |  |
| Female | 102 (60.4) | | 0.72 (0.50-1.04) | |  | | 0.75 (0.50-1.12) | |  | |  |
| Transgender | 0 (0) | | 0 | |  | | 0 | |  | |  |
| Male | 294 (67.9) | | Reference | |  | | Reference | |  | |  |
|  |  | |  | |  | |  | |  | |  |
| **HIV/AIDS Diagnosis** |  | |  | | **0.01** | |  | | **0.001** | |  |
| HIV Diagnosis | 267 (68.8) | | 1.54 (1.09-2.17) | |  | | 1.99 (1.35-2.93) | |  | |  |
| AIDS Diagnosis | 129 (58.9) | | Reference | |  | | Reference | |  | |  |
|  |  | |  | |  | |  | |  | |  |
| **Financial Status** |  | |  | | **0.04** | |  | | 0.09 | |  |
| 251-400% FPL (Tax Credit) | 34 (75.6) | | 1.92 (0.95-3.91) | |  | | 1.54 (0.72-3.30) | |  | |  |
| 139-250% FPL (Tax Credit) | 92 (73.6) | | 1.73 (1.11-2.71) | |  | | 1.77 (1.09-2.86) | |  | |  |
| 101-138% FPL      (Medicaid Gap with Tax Credit) | 32 (62.7) | | 1.05 (0.57-1.92) | |  | | 0.89 (0.46-1.73) | |  | |  |
| <100% FPL      (Medicaid Gap, no Tax Credit) | 238 (61.7) | | Reference | |  | | Reference | |  | |  |
|  |  | |  | |  | |  | |  | |  |
| **Region of Residence** |  | |  | | **< 0.001** | |  | | **< 0.001** | |  |
| Northwest | 34 (81.0) | | 1.76 (0.74-4.20) | |  | | 2.13 (0.86-5.29) | |  | |  |
| Eastern | 134 (54.9) | | 0.51 (0.32-0.81) | |  | | 0.57 (0.34-0.97) | |  | |  |
| Central | 89 (66.9) | | 0.84 (0.49-1.44) | |  | | 1.03 (0.57-1.86) | |  | |  |
| Southwest | 57 (79.2) | | 1.58 (0.79-3.16) | |  | | 2.12 (0.98-4.58) | |  | |  |
| Northern | 82 (70.7) | | Reference | |  | | Reference | |  | |  |
|  |  | |  | |  | |  | |  | |  |
| Days Observed |  | |  | | 0.1 | |  | | 0.6 | |  |
|  |  | |  | |  | |  | |  | |  |
| Abbreviations: CI: Confidence Interval, ADAP: AIDS Drug Assistance Program, QHP: Qualified Health Plan, HIV: Human Immunodeficiency Virus, AIDS: Acquired Immunodeficiency Syndrome, FPL: Federal Poverty Level. | | | | | | | | | | |  |
