# Supplementary figures and images for: Year 2 of Affordable Care Act Qualified Health Plans (QHPs) in a Medicaid Nonexpansion State: QHPs Associated With Viral Suppression for Virginia AIDS Drug Assistance Program Clients
Source: Open Forum Infect Dis. 2018 Oct 31;5(12):ofy283. doi: 10.1093/ofid/ofy283 (PMC6293482; doi:10.1093/ofid/ofy283)

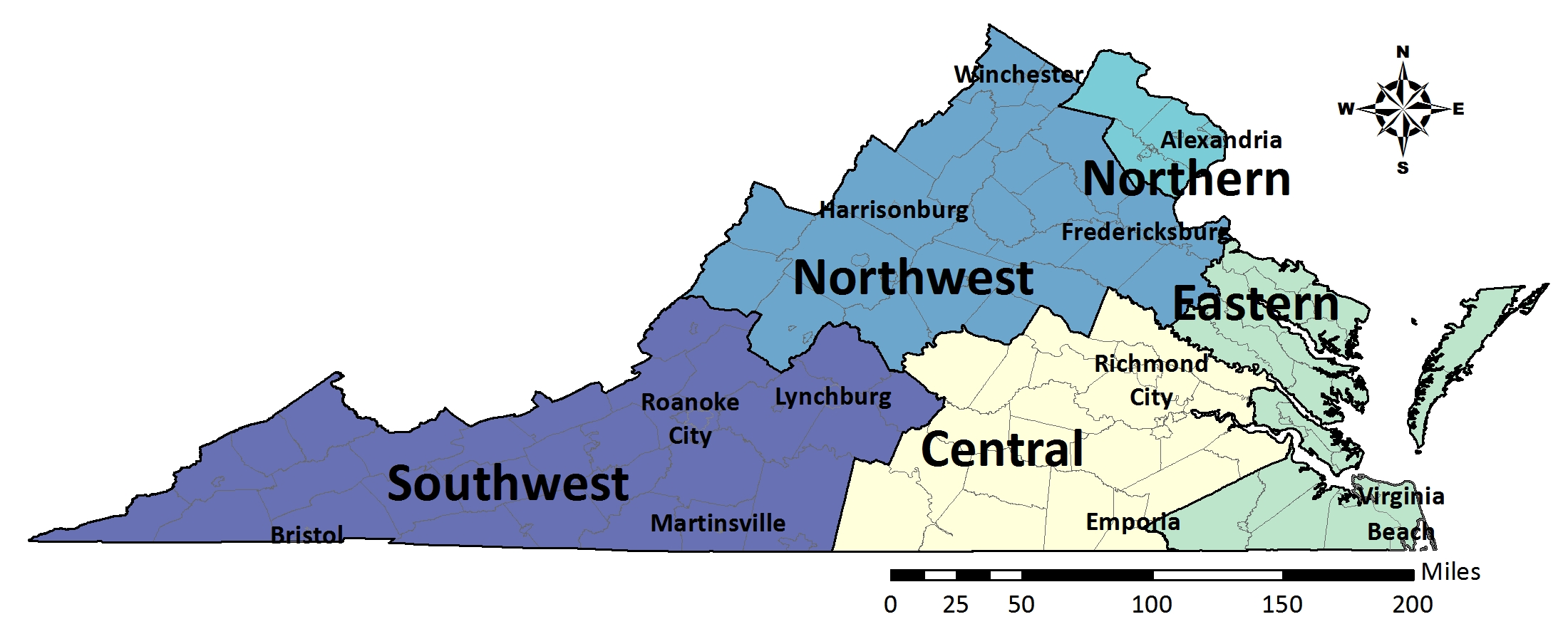

Supplement: ofy283_suppl_supplementary_figure [file ofy283_suppl_supplementary_figure.png]
